# Supplementary material for: Causal Relationship between Aging and Anorexia Nervosa: A White-Matter-Microstructure-Mediated Mendelian Randomization Analysis
Source: Biomedicines. 2024 Aug 16;12(8):1874. doi: 10.3390/biomedicines12081874 (PMC11351342; doi:10.3390/biomedicines12081874)
Supplement: Supplementary file 1 [file biomedicines-12-01874-s001.zip › Supplementary Figure/Figure S8/Leaveoneout plot of MR analysis for TL to ubm-b-1778.pdf]

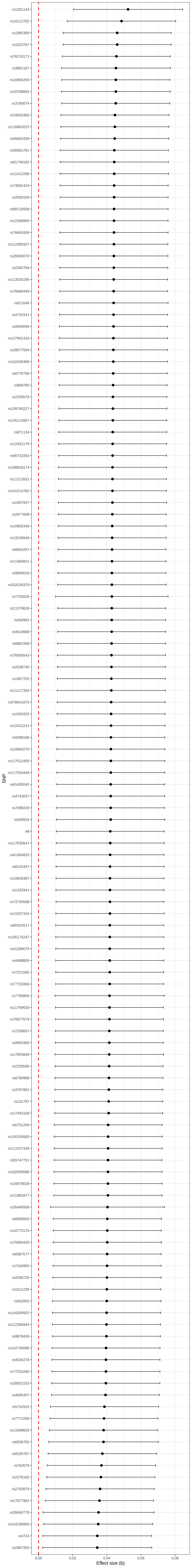
rs1291143
rs10112752
rs1985369
rs1023767
rs76219171
rs3891167
rs10905255
rs10768683
rs3785074
rs34550383
rs116863223
rs45604339
rs56061761
rs61748181
rs11412296
rs73581419
rs2555104
rs80116508
rs12369950
rs78491606
rs111950327
rs28363070
rs2282764
rs113525195
rs76666449
rs611646
rs4731541
rs9940099
rs137901416
rs28577594
rs142426306
rs6776756
rs869785
rs2293579
rs139795227
rs145114957
rs871134
rs12932179
rs66731853
rs188918174
rs11212631
rs141214782
rs1957937
rs2977608
rs10805346
rs13230646
rs6054257
rs11584821
rs9600019
rs202034370
rs7705526
rs11579626
rs450962
rs4616688
rs6881568
rs76065543
rs2538745
rs1907702
rs11117354
rs376641875
rs1003322
rs12412214
rs9398196
rs10840270
rs117512405
rs117034449
rs61405042
rs4743037
rs7099229
rs939916
All
rs117630647
rs41304832
rs8102497
rs10845387
rs1332941
rs73730598
rs11557154
rs80324517
rs185174247
rs41269079
rs4498805
rs7221585
rs77732866
rs7790856
rs11769630
rs79977579
rs7209057
rs9955360
rs17803849
rs2230590
rs6790988
rs3767952
rs131797
rs17445108
rs6751209
rs150150565
rs111527438
rs55747751
rs182059586
rs16978028
rs11991877
rs35446936
rs6669563
rs10773176
rs75664430
rs6587577
rs7164950
rs2056726
rs1611236
rs932002
rs144204502
rs112394943
rs9878436
rs142730696
rs4530278
rs77231040
rs28502153
rs4695407
rs5742915
rs7772289
rs11699829
rs6536702
rs8105767
rs762679
rs2276182
rs2763979
rs17677991
rs35640778
rs143190905
rs4724
rs2967355
SNP
0.00
0.02
0.04
0.06
0.08
Effect size (b)
